# Health Tract, No. 70

**Published:** 1865

**Authors:** 


### HEALTH TRACT, No. 70.

### DEAFNESS

Is sometimes hereditary, and runs in families; at others, the result of violent strains, as in the retching of vomiting. It often succeeds ill-cured or ill-managed attacks of scarlet-fever, measles, chicken-pox, and the like; it sometimes arises from inanition, a gradual withering away of the parts. In almost all cases, the only really useful and safe plan of treatment is to keep up the general health, and wear in the ailing ear a bit of cotton, moistened with glycerine; or let fall a drop or two in the ear; then put the cotton in; the whole object being to keep the parts moist. Glycerine is the best substance known for that object, because it is as perfectly mild and safe as milk and water, while it retains its moisture longer than any other substance known in nature; hence, if hardened "wax" causes the deafness, it will certainly be softened and brought away. Auricles, or other "aids" to hearing, improve for a time, but only to bring ultimate deafness the sooner and more certainly, as well as more completely. In ninety-nine cases out of a hundred of deafness, in the ordinary walks of life, all tinkering with the ear is worse than useless—it is pernicious; and quite as often will it be found that whatever general good health, and the constant (once daily) moistening with pure glycerine does not accomplish in the way of an improved hearing, nothing else will. The safest general rule for all is, never allow any thing to touch the eye or ear stronger than lukewarm rain-water or glycerine, (which is really oil and water,) unless by the special advice of an educated physician, whom you have long known. After all, deafness is not an unmixed evil. Multitudes would be happier if they did not hear quite so much; besides, it is a source of fun sometimes. For example:

In a town in New-Hampshire lived old Farmer P——, who was very deaf. On his farm, near the road, stood a very large tree, and thirty feet from the ground on this tree was a large knot.

As Farmer P—— was passing by one day, he thought he would cut it down to make a mill-post of. He had been at work some time, when he thought some stranger would come along and ask him the following questions and he would make the following answers:

"What is that tree for?" asks the stranger.

"A mill-post," replies the farmer.

"How long are you going to cut it?"

"Up to that knot."

"How much do you ask for it?"

"Five dollars."

"I won't give it."

"Well, if you don't, somebody else will."

As old Farmer P—— was working away, sure enough a stranger did come along, and the following dialogue ensued:

"Good morning, sir," said the stranger.

"A mill-post," replied the farmer.

"How far is it down to the corner?"

"Up to that knot."

"You don't understand me; how far is it down to that corner?"

"Five dollars."

"You old scamp! I have a good mind to give you a whipping!"

"Well, if you don't, somebody else will."

